# Weight Stigma and Orthopedic Surgeons' Treatment Preferences for Patients With Obesity Who Are Candidates for Elective Total Knee Arthroplasty

**DOI:** 10.1002/osp4.70059

**Published:** 2025-02-11

**Authors:** Yaniv Yonai, Rawan Masarwa, Merav Ben Natan, Yaron Berkovich

**Affiliations:** ^1^ The Orthopedics B Department Hillel Yaffe Medical Center Hadera Israel; ^2^ Pat Matthews Academic School of Nursing Hillel Yaffe Medical Center Hadera Israel; ^3^ Nursing Department Tel Aviv University Tel Aviv Israel

**Keywords:** orthopedic surgeons, total knee arthroplasty, treatment decisions, weight stigma

## Abstract

**Objective:**

This study aimed to examine how anti‐fat attitudes and attitudes toward obesity management influence orthopedic surgeons' treatment preferences for patients with obesity who are candidates for elective total knee arthroplasty (TKA).

**Methods:**

A cross‐sectional survey was conducted among 150 orthopedic surgeons using a web‐based questionnaire. The survey included four sections: socio‐demographic data, the Antifat Attitudes Questionnaire (AFA) assessing biases related to obesity (dislike, fear of fatness, and beliefs about willpower), an adapted questionnaire on attitudes toward obesity management, and a custom section on treatment preferences.

**Results:**

The sample had a mean age of 43.4 years (SD = 9.7) and was predominantly male (70.7%). Participants exhibited moderate anti‐fat attitudes alongside positive views on obesity management. Stronger anti‐fat attitudes correlated with a preference for conservative treatments over surgery (*r* = 0.45 to *r* = 0.29, *p* < 0.001), whereas supportive attitudes toward obesity management were associated with less preference for conservative treatment (*r* = −0.53, *p* < 0.001). Male surgeons demonstrated higher anti‐fat attitudes and a greater inclination for conservative treatment than female surgeons. Regression analysis identified attitudes toward obesity management as a significant predictor of treatment preferences (*β* = −0.54, *p* < 0.001).

**Conclusion:**

Findings highlight the impact of weight stigma on clinical decision‐making and emphasise the need for increased awareness and education to ensure equitable access to TKA for patients with obesity.

## 1 Introduction

Obesity is a serious health problem associated with negative attitudes and biases in modern society. Modern society has adopted negative stereotypes toward individuals with obesity, for instance, that individuals with obesity are irresponsible, lazy, and lack self‐discipline [1, 2]. Weight stigma is the social devaluation and rejection of individuals who do not conform to societal standards of what is considered an ideal body weight. This social stigma is often accompanied by negative weight‐based stereotypes, such as the belief that individuals with obesity are lazy or lack self‐discipline [3]. Weight stigma can lead to social exclusion, marginalisation, and discrimination [4].

Weight stigma is particularly pervasive in healthcare; it can have serious health and behavioral consequences, such as increased stress, mental health issues, and avoidance of healthcare services [5, 6, 7], with significant negative consequences for the quality of care provided to patients with obesity. For example, patients with obesity may avoid seeking follow‐up care because of fear of judgment or mistreatment, leading to worse health outcomes [8, 9, 10]. There is evidence that the treatment of patients with obesity differs from those without obesity. For example, Rathbone et al. investigated physicians' hypothetical approach to a patient with obesity seeking care for migraines [11]. They observed that greater endorsement of weight stigma among professionals was associated with a higher focus on the patient's weight than on their migraines. Another example is the study by Alessi et al., which revealed that patients with type 2 diabetes and obesity more frequently failed to receive pharmacological treatment, when necessary, than patients without obesity. They suggested that weight stigma was suggested as a possible factor in delaying the intensification of treatment in these patients [12].

There is substantial evidence indicating that patients with obesity, particularly those suffering from knee osteoarthritis or knee injuries, may benefit from total knee arthroplasty (TKA), a common surgical intervention for individuals suffering from knee pain, particularly those with osteoarthritis, a condition that often worsens due to excess body weight [13, 14, 15]. Obesity is recognised as a major risk factor for knee osteoarthritis due to the increased mechanical stress placed on the joints, as well as the inflammatory effects of adiposity. Excess body weight not only accelerates the degeneration of joint cartilage but also increases the likelihood of chronic pain and disability, often leading to the consideration of TKA as a treatment option [14]. However, the benefits of surgery in these patients must be carefully weighed against the increased risk of peri‐operative complications, including higher rates of infection, wound healing problems, and deep vein thrombosis, which are more prevalent among individuals with obesity [16, 17, 18]. Additionally, studies have shown that patients with obesity may experience less favorable outcomes post‐surgery, such as slower recovery times and increased need for post‐operative care [15, 16].

The decision to proceed with TKA in patients with obesity is often influenced by the patient's BMI, and this can be complicated by weight stigma within the medical community. The literature suggests that weight stigma among orthopedic surgeons may affect their treatment preferences. For instance, Hill et al. reported that a significant proportion of orthopedic surgeons impose BMI thresholds for TKA, potentially delaying necessary procedures based on weight alone, which may reflect weight stigma [19]. Similarly, Godziuk et al. highlighted that orthopedic surgeons frequently expect patients to lose weight as evidence of their commitment, a requirement not typically imposed on patients with other risk factors [20].

Despite these observations, there is a dearth of studies investigating the potential effect of weight stigma on treatment decisions among orthopedic surgeons. Therefore, this study aimed to fill this gap by exploring the role of anti‐fat attitudes and attitudes regarding obesity and its management in orthopedic surgeons' treatment preferences for patients with obesity who were candidates for elective TKA.

## 2 Materials and Methods

The study employed a cross‐sectional survey design. A convenience sample of 150 orthopedic surgeons participated in the study. Data were collected through an online questionnaire consisting of several sections. The first section gathered socio‐demographic and professional data using nine items. The second section utilized the Antifat Attitudes Questionnaire (AFA) [21]. The AFA was a multi‐factorial questionnaire consisting of 13 items. The first factor, termed ''dislike'', comprised seven items and assessed feelings toward individuals with overweight and/or obesity (e.g., ''Overweight people should not be employed''). The second factor, ''fear of fat'', consisted of three items and evaluated the fear of gaining weight (e.g., ''Gain weight is one of the worst things''). Lastly, the third factor, ''willpower'', included three items measuring the belief in the controllability of obesity (e.g., ''Overweight people are responsible for their weight''). Participants were asked to rate each item on a 10‐point Likert scale (from 0–completely disagree to 9–completely agree), with higher scores within each factor indicating elevated levels of anti‐fat attitudes. Mean scores for each factor were categorized into low, moderate, and high levels. For simplicity, the range of possible scores (0–9) was divided into three equal intervals: low (0.00–2.99), moderate (3.00–5.99), and high (6.00–9.00). In this research, the internal reliability (Cronbach's *α*) for anti‐fat attitudes was 0.82.

The next section of the study utilized a questionnaire adapted from Bocquier's instrument, originally designed to assess General Practitioners' attitudes regarding obesity and its management [22]. The questionnaire was appropriately modified to align with the context of orthopedic surgeons. It included 10 items (e.g., "Normal weight is important for health" and "I feel well prepared to manage overweight and obese patients"). Participants were asked to rate each item on a six‐point Likert scale (from 1–completely disagree to 6–completely agree), with higher scores reflecting a stronger endorsement of attitudes supportive of addressing obesity as a healthcare concern, as well as a heightened dedication to promoting the health and well‐being of individuals affected by obesity. Mean scores for this factor were categorized into low, moderate, and high levels. For simplicity, the range of possible scores (1–6) was divided into equal intervals: low (1.00–2.66), moderate (2.67–4.33), and high (4.34–6.00) endorsement of attitudes supportive of addressing obesity as a healthcare concern, as well as a heightened dedication to promoting the health and well‐being of individuals affected by obesity. The internal reliability for this part was Cronbach's *α* = 0.90.

The final section was designed by the researchers for the purpose of this research. This part investigated orthopedic surgeons' treatment preferences for patients with obesity who are candidates for elective TKA (BMI ≥ 30 kg/m^2^). This part included five items (e.g., ''I would prefer to postpone surgery for this patient'' and ''I would prefer conservative treatment over surgical intervention''). Participants were asked to rate each item on a six‐point Likert scale, ranging from 1 (completely disagree) to 6 (completely agree). Higher scores on this scale indicated a stronger preference for conservative treatment over surgical intervention within this patient population.

The range of possible mean scores for the treatment preference questionnaire (1–6) was divided into three equal intervals to classify levels of preference. Scores from 1.00 to 2.66 were categorized as low, indicating a weak preference for conservative treatment over surgical intervention. Scores from 2.67 to 4.33 were classified as moderate, reflecting a balanced or neutral stance on treatment preference. Lastly, scores from 4.34 to 6.00 were categorized as high, representing a strong preference for conservative treatment over surgical intervention in patients with obesity who are candidates for elective TKA. The content validity of this section was confirmed by three orthopedic surgery experts, and its internal reliability was Cronbach's *α* = 0.85.

The questionnaire was distributed among orthopedic surgeons who attended an orthopedics conference. A link to the questionnaire was sent to the participants' cellular phones during a break. The questionnaire was accompanied by an explanation of the purpose of the study, and voluntary participation was emphasized. Anonymity was assured. Completion of the questionnaire was considered consent to participate in the study. The questionnaire was sent to 160 orthopedic surgeons and 150 questionnaires were completed, with a response rate of 94%. No incentives were provided to the research participants. The research was reported in line with the STROCSS 2021 guidelines [23].

Statistical analysis was conducted using IBM SPSS Statistics (version 28.0, IBM Corp., Armonk, NY, USA). Descriptive statistics were utilized to outline sample characteristics. Associations between variables were assessed using independent samples t‐tests, Chi‐square tests, and Pearson's correlation coefficients. Additionally, linear regression analysis was employed to identify predictors of orthopedic surgeons' attitudes toward treatment decisions for patients with obesity. Statistical significance was set at *p* < 0.05. This study was performed in line with the principles of the Declaration of Helsinki. Ethical approval for the research was provided by the institutional IRB.

## 3 Results

One hundred and fifty orthopedic surgeons took part in the study. The mean age of the participants was 43.4 (SD = 9.7, range 25–72). Most were male (70.7%, *n* = 106). Slightly more than half of the sample (56.7%, *n* = 85) were board‐certified orthopedic surgeons, while 43.3% (*n* = 65) were orthopedic residents/fellows. The mean tenure of the participants was 9.04 years (SD = 6.3, range 1–30).

Table 1 outlines the distribution of the study variables, highlighting a moderate level of anti‐fat attitudes among orthopedic surgeons. These attitudes were reflected in various dimensions. For instance, 30% of the surgeons believed that fat individuals should not be employed, while an equal proportion disagreed, illustrating a divided stance on this matter. Similarly, moderate concerns about weight gain (fear of fat) were observed, with 22% agreeing that gaining weight is among the worst things that could happen, contrasted by another 22% who disagreed, showing a split in opinions. Furthermore, attitudes toward personal responsibility for weight were also moderate, as 28% of surgeons believed that individuals are solely responsible for their weight, while an equivalent percentage opposed this view, underscoring a varied perspective on the issue.

**TABLE 1:** Distribution of the study variables (*N* = 150).

	Mean	Standard deviation	Minimum	Maximum
Anti‐fat attitudes				
Dislike	3.7	1.8	1.00	8.83
Fear of fat	3.7	1.9	1.00	9.00
Willpower	3.8	2.0	1.00	9.00
Attitudes regarding obesity and its management	3.9	1.0	1.67	5.75
Treatment preferences	4.5	0.90	2.20	6.00

In addition, orthopedic surgeons demonstrated moderately positive attitudes toward obesity as a healthcare concern and its management. Specifically, the surgeons generally supported addressing obesity within the healthcare context and expressed a moderate commitment to promoting the health and well‐being of individuals affected by obesity. For instance, 65% of surgeons reported feeling well‐prepared to manage overweight and obese patients, while 25% indicated they did not feel adequately prepared, reflecting some variability in confidence levels.

Regarding treatment preferences for patients with obesity, orthopedic surgeons showed a moderate to high preference for conservative treatment over surgical interventions. For example, 58% of surgeons agreed that they would prefer conservative treatment approaches, such as physical therapy or weight management programs, over TKA for patients with obesity, whereas 32% expressed a preference for surgical intervention, highlighting differing approaches to patient care.

Positive associations were found between dislike (*r* = 0.45, *p* < 0.001), fear of fat (*r* = 0.35, *p* < 0.001), willpower (*r* = 0.29, *p* < 0.001), and treatment preferences for patients with obesity. That is, the stronger the orthopedic surgeons' explicit antipathy toward fat people, as well as their concerns and distress about weight or the prospect of becoming overweight and their belief that being overweight is a matter of personal control or lack thereof–the stronger their preference for conservative treatment over surgical intervention.

In addition, a negative association was found between orthopedic surgeons' attitudes regarding obesity and its management, and their treatment preferences for patients with obesity (*r* = −0.53, *p* < 0.001). That is, the more the orthopedic surgeons endorse attitudes supportive of addressing obesity as a healthcare concern and demonstrate dedication to promoting the health and well‐being of individuals affected by obesity, the less pronounced their preference for conservative treatment over surgical intervention in patients with obesity. Of note, the orthopedic surgeons' age was not found to be associated with their treatment preferences.

A statistically significant difference was found between male and female orthopedic surgeons in their treatment preferences for patients with obesity (*t* = 2.2 [df = 66.54], *p* < 0.05). Namely, male orthopedic surgeons expressed a greater preference for conservative treatment over surgical intervention (*M* = 4.6, SD = 0.8) than did female orthopedic surgeons (*M* = 4.2, SD = 1.0). In addition, a difference of borderline statistical significance was found between male and female orthopedic surgeons in the dislike variable (*t* = 1.8 [df = 148], *p* = 0.067). Namely, male orthopedic surgeons tended to express more explicit antipathy toward fat people (*M* = 3.9, SD = 1.8) than did female orthopedic surgeons (*M* = 3.3, SD = 1.6). In contrast, no differences were found between board‐certified orthopedic surgeons and orthopedic residents/fellows in any of the study variables.

Dislike, fear of fat, willpower, attitudes regarding obesity and its management, and gender were entered into a linear regression, to test whether these variables explain orthopedic surgeons' treatment preferences for patients with obesity. The results of the regression indicated that only attitudes regarding obesity and its management significantly predicted treatment preferences (*β* = −0.54, *p* < 0.001).

## 4 Discussion

This study examined the relationship between orthopedic surgeons' treatment preferences for individuals with obesity who are candidates for elective TKA and two key factors: anti‐fat attitudes and attitudes regarding obesity and its management. In this study, participants tended to express a moderate level of anti‐fat attitudes. These findings align with prior research suggesting the presence of weight stigma among orthopedic surgeons [19, 20].

The study also revealed, however, that the anti‐fat attitudes were not pronounced, which could be attributed to changing social norms regarding the acceptability of expressing negative attitudes toward individuals with obesity [24], and to intervention programs aimed at developing sensitivity and empathy among healthcare professionals toward patients with obesity.

In this study, positive associations were found between anti‐fat attitudes and treatment preferences for individuals with obesity. That is, the stronger the orthopedic surgeons' explicit antipathy toward individuals with obesity, their concerns and distress about weight or the prospect of becoming overweight, and their belief that being overweight is a matter of personal control or lack thereof–the stronger their preference for conservative treatment over surgical intervention in individuals with obesity. These findings confirmed the assumption that weight stigma may affect orthopedic surgeons' treatment decisions [19, 20] and were consistent with other studies that suggest an association between weight stigma and physicians' treatment decisions [11, 12]. More precisely, weight stigma may lead orthopedic surgeons to prefer conservative treatment over surgical intervention in individuals with obesity. These findings are concerning, as certain patients may be denied the opportunity to benefit from surgery.

Moreover, a negative correlation emerged between orthopedic surgeons' attitudes regarding obesity management and their treatment preferences for individuals with obesity. This indicates that as orthopedic surgeons increasingly endorse attitudes supportive of addressing obesity as a disease and exhibit dedication to promoting the health and well‐being of individuals affected by obesity, they are less inclined to favor conservative treatment approaches when managing patients with obesity, possibly opting for surgical interventions instead. These findings imply that orthopedic surgeons' treatment preferences for patients with obesity may also be influenced by their awareness and desire to promote well‐being in these patients. Importantly, attitudes regarding obesity management were identified as predictors of treatment preferences, whereas anti‐fat attitudes were not.

In the present study, male orthopedic surgeons tended to express more explicit antipathy toward fat people than did female orthopedic surgeons. This finding is consistent with previous research indicating that males have more negative attitudes toward individuals with obesity than females [24]. This gender difference has also been documented among physicians [6]. One suggested explanation for this difference is the internalization‐externalization hypothesis, which states that women internalize the value of slimness as presented in the mass media, while men, in contrast, externalize it. That is, women tend to focus on their weight, while men tend to focus on other people's weight, and consequently have a greater dislike of fat people [25].

In this study, male orthopedic surgeons expressed a greater preference for conservative treatment over surgical intervention in patients with obesity than female orthopedic surgeons. This finding may be explained by the fact that in the present study, male orthopedic surgeons scored higher on dislike, while higher dislike was associated with a stronger preference for a conservative treatment.

In this study, an orthopedic surgeon's age, professional qualification (i.e., board‐certified orthopedic surgeons vs. orthopedic residents/fellows), and tenure were not found to be associated with their attitudes toward treatment decisions for patients with obesity. These findings possibly reflect the strong influence of the surrounding society regarding obesity and individuals with obesity, which may blur the potential impact of any other factors [26, 27].

Several limitations should be considered when interpreting the findings. First, the use of convenience sampling may introduce selection bias and limit the generalizability of the study results to a broader population of orthopedic surgeons. Participants who voluntarily participated in the study may differ systematically from those who did not, potentially affecting the representativeness of the sample. Additionally, the reliance on self‐reported measures, particularly regarding sensitive topics such as attitudes toward obesity and its management, may be susceptible to social desirability bias. Participants may provide responses that they perceive as socially acceptable rather than reflecting their true attitudes. Furthermore, the cross‐sectional nature of the study design precludes the establishment of causal relationships between variables.

In conclusion, this study suggests that anti‐fat attitudes and particularly the attitudes regarding obesity and its management, seem to have the potential to affect treatment decisions, namely, to lead orthopedic surgeons to prefer conservative over surgical treatment in patients with obesity who are candidates for elective TKA. These findings underscore the importance of addressing weight stigma and promoting awareness of obesity as a healthcare concern among orthopedic surgeons. Interventions aimed at mitigating anti‐fat attitudes and fostering a patient‐centered approach to obesity management, such as using person‐first language, addressing misconceptions about obesity, providing additional education on the disease model of obesity, and promoting empathetic communication, may help optimize treatment decisions and ensure equitable access to surgical interventions for individuals with obesity. Additionally, efforts to promote gender‐sensitive interventions may be warranted, given the observed gender differences in anti‐fat attitudes and treatment preferences. Ultimately, by addressing weight stigma and fostering a supportive and empathetic approach to obesity management, orthopedic surgeons can play a vital role in improving outcomes and promoting health equity among individuals with obesity.

## Author Contributions

**Yaniv Yonai:** conceptualization, methodology, validation, writing–original draft, writing–review & editing. **Rawan Masarwa:** methodology, validation, data curation, writing–original draft, writing–review & editing. **Merav Ben Natan:** methodology, validation, formal analysis, writing–original draft, writing–review & editing, visualization. **Yaron Berkovich:** data curation, writing–original draft, writing–review & editing.

## Ethics Statement

This study was conducted in line with the principles of the Declaration of Helsinki. Approval was granted by the Hillel Yaffe Medical Center (Date 2022./No129).

## Consent

Informed consent was secured from all individuals who completed the questionnaire.

## Conflicts of Interest

The authors declare no conflicts of interest.

5

## Data Availability

Data used in this study will be made available to other researchers or interested parties upon request.
